# Relative qPCR to quantify colonization of plant roots by arbuscular mycorrhizal fungi

**DOI:** 10.1007/s00572-020-01014-1

**Published:** 2021-01-21

**Authors:** Natacha Bodenhausen, Gabriel Deslandes-Hérold, Jan Waelchli, Alain Held, Marcel G. A. van der Heijden, Klaus Schlaeppi

**Affiliations:** 1 Plant Soil Interactions, Department of Agroecology and Environment, Agroscope, Zurich, Switzerland; 2 Department of Soil Sciences, Research Institute of Organic Agriculture FiBL, Frick, Switzerland; 3 Institute of Plant Sciences, University of Bern, Bern, Switzerland; 4 Plant Microbe Interactions, Department of Environmental Sciences, University of Basel, Basel, Switzerland; 5 Department of Plant and Microbial Biology, University of Zürich, Zurich, Switzerland

**Keywords:** Quantitative PCR (qPCR), Arbuscular mycorrhizal fungi, Relative quantification, *Petunia*

## Abstract

Arbuscular mycorrhiza fungi (AMF) are beneficial soil fungi that can promote the growth of their host plants. Accurate quantification of AMF in plant roots is important because the level of colonization is often indicative of the activity of these fungi. Root colonization is traditionally measured with microscopy methods which visualize fungal structures inside roots. Microscopy methods are labor-intensive, and results depend on the observer. In this study, we present a relative qPCR method to quantify AMF in which we normalized the AMF qPCR signal relative to a plant gene. First, we validated the primer pair AMG1F and AM1 in silico, and we show that these primers cover most AMF species present in plant roots without amplifying host DNA. Next, we compared the relative qPCR method with traditional microscopy based on a greenhouse experiment with *Petunia* plants that ranged from very high to very low levels of AMF root colonization. Finally, by sequencing the qPCR amplicons with MiSeq, we experimentally confirmed that the primer pair excludes plant DNA while amplifying mostly AMF. Most importantly, our relative qPCR approach was capable of discriminating quantitative differences in AMF root colonization and it strongly correlated (Spearman Rho = 0.875) with quantifications by traditional microscopy. Finally, we provide a balanced discussion about the strengths and weaknesses of microscopy and qPCR methods. In conclusion, the tested approach of relative qPCR presents a reliable alternative method to quantify AMF root colonization that is less operator-dependent than traditional microscopy and offers scalability to high-throughput analyses.

## Introduction

The majority of terrestrial plant species form a symbiotic relationship with arbuscular mycorrhizal fungi (Smith and Read 2010; van der Heijden et al. 2015). These fungi belong to the phylum Glomeromycota, and they mainly provide the plant with macronutrients such as phosphorus (P) and nitrogen (N) as well as micronutrients (iron, copper, and zinc) in exchange for carbohydrates and lipids (Walder and van der Heijden 2015; Keymer and Gutjahr 2018). Arbuscular mycorrhizal fungi (AMF) form characteristic arbuscules when colonizing the cortices of plant roots, and they develop a dense network of hyphae in the soil, spreading well beyond roots and thus increasing the volume of soil explored for nutrients. The extent to which plant roots are colonized by AMF is affected by many factors including agricultural practices (Jansa et al. 2006), soil parameters such as the amount of phosphate available to plants (Kahiluoto et al. 2001), and plant genetics (Parniske 2008). The level of root colonization provides an indication of the abundance, growth, and activity of AM fungi (Smith and Read 2010). Therefore, it is of key importance that AMF root colonization can be quantified precisely.

AMF root colonization is traditionally measured with the microscope or sometimes using phospholipid fatty acid analysis (PLFA). For PLFA, fatty acids are extracted from plant roots, analyzed by gas chromatography coupled with mass spectrometry, and the phospholipid 16:1ω5 often is used as biomarker for AMF (Olsson et al. 1995). Although 16:1ω5 represents a major fraction of the total phospholipids in AMF (Olsson et al. 1998), this biomarker also is present in other microorganisms, and concentrations differ among different AMF species (Graham et al. 1995), thus confounding the analysis (Kirk et al. 2004).

Microscopy-based methods rely on preceding cytological staining of AMF structures. Root samples are first cleared in boiling KOH and then are stained using stains such as common ink (Vierheilig et al. 1998) or trypan blue (Phillips and Hayman 1970). For microscopic quantification, the two most-used methods are the so-called “grid-line intersect” (Giovanetti and Mosse 1980) and the “magnified intersection” (McGonigle et al. 1990) protocols. The “grid-line intersect” method relies on a simple dissecting microscope (often with magnifications from 7x to 50x) used to observe roots placed in a petri dish with grid lines that guide assessments. While a relatively large amount of the root system can be examined with this method, a disadvantage is that fungal structures such as arbuscules might not be recognized at this low level of magnification. The “magnified intersection” method solves this limitation with small root fragments that are carefully mounted on microscopy slides and examined with a light microscope (often with magnifications from 20x to 250x). It is worth mentioning that meticulous preparation of the microscopy slides is time-consuming. For both microscopy methods, approximately 100 intersections per sample are counted for accurate quantification of AMF root colonization. This scoring of intersections is time-consuming, tedious, and can be highly dependent on the observer. Beginners need to be well-trained by experienced researchers for accurate identification of the different fungal structures. Despite these limitations, AMF scoring by microscopy is a well-established and reliable approach to quantify the level of root colonization as well as to obtain insights into fungal physiology by scoring fungal structures.

In the past two decades, various types of quantitative PCR (qPCR) approaches have been developed to quantify AMF (Janoušková and Caklová 2020). Approaches differ by the type of target primers, type of fluorescence technique, and type of data normalization. Quantitative PCR primers may target single AMF species including *Rhizophagus intraradices* (formerly: *Glomus intraradices*), *Funneliformis mosseae* (formerly: *Glomus mosseae*), *Claroideoglomus claroideum* (formerly: *Glomus claroideum*), *Glomus aggregatum*, or *Gigaspora margarita* (Alkan et al. 2004, 2006; Isayenkov et al. 2004; Jansa et al. 2008; Gamper et al. 2008; Thonar et al. 2012; Knegt et al. 2016). Alternatively, qPCR primers were designed to capture a broad diversity of AMF (Hewins et al. 2015) so that all species of a mycorrhizal community in a root can be simultaneously quantified. This particular approach relies on the reverse primer AM1 (Helgason et al. 1998), which was designed to amplify fungal sequences and exclude plant sequences, and AMG1F (Hewins et al. 2015), which also avoids amplification of plant DNA (*Allium tricoccum*, the plant species of interest in that study). The two primers bind to the small subunit of the ribosomal operon (also called the 18S rRNA gene), which was identified as a suitable marker region for the quantification of multiple AMF species because it relates well to fungal biomass (Voříšková et al. 2017). AMF qPCR approaches further differ by fluorescence technique, either being dye-based (Alkan et al. 2004; Jansa et al. 2008; Werner and Kiers 2015; Hewins et al. 2015) or probe-based (Gamper et al. 2008; König et al. 2010; Thonar et al. 2012). In both techniques, AMF-specific primers produce double-stranded DNA amplicons while their quantification differs depending on whether the fluorescence signal results from intercalation of a dye or from a probe that was hydrolyzed during amplification. Probe-based approaches are highly specific because the hydrolysis probe ensures a third annealing to the target DNA (besides the binding of the two qPCR primers). Dye-based approaches have the key advantage that the fluorescence signal can be experimentally validated by sequencing the resulting amplicons. Finally, qPCR approaches differ by their type of data normalization – absolute vs. relative quantification (see paragraph below). While several of the developed qPCR approaches that target single AMF species successfully have been cross-validated with microscopy or PLFA methods (Alkan et al. 2004; Isayenkov et al. 2004; Gamper et al. 2008), to our knowledge, experimental validation has not been done for the “whole-community AMF” qPCR approach by Hewins et al. (2015).

Most AMF qPCR applications rely on absolute quantification (Alkan et al. 2004; Gamper et al. 2008; Thonar et al. 2012), a data normalization method that often is used in environmental studies (Brankatschk et al. 2012). Absolute quantification builds on a standard curve, where a known concentration of template DNA (either PCR product, plasmid with cloned insert, or genomic DNA) is serially diluted. The fluorescence signal in a sample is then translated to an absolute amount of AMF DNA based on the standard curve, which represents a linear regression of the log concentration of the standard DNA vs Ct (numbers of cycles to reach the threshold of fluorescence). Such absolute amounts are expressed as ng genomic DNA per mg root dry or fresh weight, or alternatively, absolute amounts can be normalized per volume (µl) or per total amount of DNA in the extracts. Hence, the final data transformations rely on highly accurate sample weighing (often in the range of mg) or accurate pipetting of small volumes (µl) during DNA extractions and qPCR.

Relative quantification, also referred to as the **ΔΔ**ct method, is an alternative qPCR normalization method that is common in gene expression studies (Schmittgen and Livak 2008). Relative quantification calculates an expression ratio of a target gene normalized to a reference gene in the same sample (Pfaffl 2001). Although target and reference genes often differ in their amplification efficiencies (*E* values), relative calculations take differences in efficiency of individual qPCR primer pairs into account (Pfaffl 2001). The key advantage of relative quantification is that there is no need for a well-matched DNA standard which is impossible to define for mixtures of unknown microbes (Brankatschk et al. 2012). Moreover, relative quantification does not rely on highly accurate weighing of a root aliquot as is necessary for absolute quantification.

For our research—and, probably this applies to many other researchers in this field—we were interested in a qPCR method that permits quantification of AMF root colonization in a more high-throughput and less operator-dependent manner than classical microscopy. In this study, we benchmarked the qPCR primers of Hewins et al. (2015) by comparison to a microscopic analysis of the same samples from a greenhouse experiment with *Petunia* plants of varied degrees of AMF root colonization. We investigated the use of relative quantification with the primers AMG1F and AM1 (Hewins et al. 2015), and we chose the Glyceraldehyde 3-phosphate dehydrogenase (GAPDH), a common single-copy reference for gene expression analysis, to normalize the AMF qPCR signal to a plant qPCR signal. Furthermore, with MiSeq sequencing we confirmed that this primer pair amplifies mostly Glomeromycota DNA while avoiding amplification of plant DNA. We show that under the tested conditions our qPCR approach is AMF specific (without quantifying other fungi or plant DNA) and quantitative (discriminating different levels of AMF root colonization), and thus provides an alternative molecular method to traditional microscopy.

## Material and methods

### In silico primer analysis

Consensus sequences from the ribosomal operon of 39 AMF species were retrieved from AMF reference data (Krüger et al. 2012) and from GenBank for the sequence of the 18S rRNA gene of *Petunia axillaris* (AJ236020.1). Local alignments with the qPCR primers were performed using the Clustal Omega online tool (Sievers et al. 2011). The sequences of the AMF reference set are nearly full length rRNA operon sequences from single, cleaned AMF spores and built as “consensus sequence” from up to 10 sequence variants of each AMF isolate, thus leading to the potential presence of variable nucleotides (aka DNA wobbles) in the consensus sequences.

### Pot experiment

The pot experiment was described in a previously published study (Bodenhausen et al. 2019). The article presented fungal amplicon sequencing of root samples from both *Petunia* (line V26) and *Arabidopsis*. In parallel, the *Petunia* line W155, commonly used as a background for mutants, was grown under the same conditions but not used for microbiota analysis. Briefly, soil was collected from an agricultural grassland field site, sieved, and mixed 1:1 volume with sterilized quartz-sand. Pre-grown seedlings were transferred to 400-ml pots filled with this soil mix. Plants were grown under long-day conditions (16-h photoperiod) at 25 °C and 60% relative humidity. Plants were fertilized with a nutrient solution (Reddy et al. 2007) containing one of three concentrations of phosphate: 0.03 mM KH_2_PO_4_ (low P), 1 mM KH_2_PO_4_ (medium P), and 5 mM KH_2_PO_4_ (high P). Each plant received 250 ml of the solution over the last 6 weeks of growth before harvest.

Samples were harvested at 10 weeks. Roots were separated from the shoot with a clean scalpel. Roots were shaken to remove loosely attached soil and were washed three times in phosphate-buffered saline (137 mM NaCl, 2.7 mM KCl, 8 mM Na2HPO_4_, and 1.5 mM KH_2_PO_4_, pH 7.0; approximately 10 ml for 1 g of root fresh weight). After washing, the root fragments were cut into small pieces, mixed, and split into two equivalent subsamples of similar root thicknesses. Samples for DNA extraction were stored at − 80 °C until processing. Samples for microscopy were stored in 50% ethanol until staining. Root colonization was determined using the magnified intersection method (McGonigle et al. 1990). Roots were stained with pen ink, mounted on a microscope slide, and then examined with a light microscope. Approximately 30 cm of roots (about 20 pieces of about 1.5 cm) per sample were mounted on each slide. One hundred intersections were counted per sample: each intersection was counted as “negative”, “arbuscule”, “vesicle”, or “internal hyphae only”. Colonization is the percent of non-negative intersections.

DNA extraction was described (Bodenhausen et al. 2019). Briefly, roots were lyophilized and ground, and DNA was extracted with the NucleoSpin Soil kit (Macherey-Nagel, Düren, Germany). DNA was quantified using a Quant-iT Picogreen dsDNA Assay Kit (Invitrogen, Eugene, OR USA) on a Varian Cary Eclipse fluorescence spectrometer (Agilent Technologies, Santa Clara, CA USA) and diluted to 10 ng/μl. In the present study, we used those DNA extracts of the earlier study for validation of relative qPCR with the primers AMG1F and AM1 to quantify AMF root colonization. We also compare the new qPCR results with the previously determined levels of AMF root colonization by microscopy.

### qPCR

The reaction volumes were 20 µl and contained onefold HOT FIREPol EvaGreen qPCR Mix Plus (Solis Biodyne, Tartu, Estonia), 250 nM of each primer, 0.3% bovine serine albumin, and approximately 10 ng of root DNA. The AMF community was quantified based on the 18S rRNA gene fragment, amplified with the primers AMG1F (Hewins et al. 2015) and AM1 (Helgason et al. 1998). The 18S rRNA gene signal was normalized to the plant gene “glyceraldehyde-3-phosphate dehydrogenase,” as amplified with newly designed PCR primers GAP_f1 (5′-TGGAATGGCCTTCAGAGTTC-3′) and GAP_r3 (5′-TCTGTGGAAACCACATCGTC-3′). No-template controls were included in each run. qPCR assays were run in triplicate on a CFX96 Real Time System (Bio Rad, Hercules, California). The PCR program consisted of an initial denaturation step of 15 min at 95 °C, followed by 45 cycles of denaturation at 95 °C for 15 s, annealing at 62 °C for 30 s, and elongation at 72 °C for 20 s followed by a melting curve analysis (from 65 to 95°C, with 0.5 °C steps holding for 5 s). The raw data were exported directly from Bio-Rad CFX Manager 3.1 and imported into LinRegPCR version 2016.0 (Ruijter et al. 2009) to determine cycle number to threshold (Ct) and efficiency (*E*) using the default baseline threshold from LinRegPCR. The 18S rRNA gene signal from AMF was normalized to the plant gene signal and calculated as follows: 18S rRNA/plant gene = *E*_plant gene_^Ct plant gene^/*E*_18S_^Ct 18S^, where Ct is the mean of the 3 technical replicate reactions and *E* is the mean for all reactions with a particular primer pair for each run (Bodenhausen et al. 2014).

### Sequencing and bioinformatics

The amplicons of qPCR reactions of 12 samples (Table S1) were prepared for MiSeq sequencing. Triplicate qPCR reactions were pooled, purified with the Gel and PCR Clean-up kit (Macherey–Nagel, Dürren, Germany), quantified as before, and diluted to 1 ng/μl. Barcodes were added with a second PCR (Table S1). Reaction volumes were 20 μl with 1 × 5PRIME HotMasterMix (Quantabio, Beverly, MA, USA), 250 nM of each primer, and 0.3% bovine serine albumin. The PCR program consisted of an initial denaturation step of 3 min at 94 °C, 10 cycles of denaturation at 94 °C for 45 s, annealing at 63 °C for 30 s, elongation at 65 °C for 90 s, and final elongation at 65 °C for 10 min. After clean-up with an Agencourt AMPure XP kit (Beckman Coulter, Brea, CA, USA), DNA was quantified and pooled in equimolar fashion. Finally, DNA was concentrated with the Agencourt AMPure kit and quantified with a Qubit dsDNA HS assay on Qubit 2 fluorometer (Invitrogen, Eugene, OR, USA) and combined with other libraries for sequencing. The final library preparation was performed according to a published protocol (Bodenhausen et al. 2019) and sequenced at the Functional Genomic Center Zurich on the Illumina MiSeq Personal Sequencer. We typically sequence several different experiments in a single MiSeq run and the qPCR products of this study were included in a run of which the sequences were deposited previously (Hu et al. 2018). The raw sequencing data are available from the European Nucleotide Archive (http://www.ebi.ac.uk/ena) with the sample ID SAMEA103939171 under the study accession PRJEB20127. The sequences of the qPCR samples can be extracted from the raw data based on the barcodes and primers indicated in the mapping file (Table S1).

The raw read data were quality checked with FastQC (Andrews 2010) and demultiplexed using cutadapt (Martin 2011). We then largely followed the DADA2 pipeline from Callahan et al. (2016) using the R package dada2 (v3.10). Instead of clustering the sequences in operational taxonomic units (OTUs), the DADA2 pipeline produces amplicon sequence variants (ASVs), which replace OTUs as the units of analysis (Callahan et al. 2017). The sequences were quality filtered (max. expected errors: 0, min length: 140 bp, discard reads that match phiX), truncated (after 140 bp or at the first instance of a quality score ≤ 2), and dereplicated. A parametric error model was learned by the DADA algorithm to correct sequencing errors. Next, forward and reverse reads were merged based on identical overlap sequences of at least twelve bases. Finally, a count table was constructed, chimeras were removed, and taxonomies assigned with a naive Bayesian classifier using the DADA2 formatted 18S training set (silva_132.18s.99_rep_set.dada2.fa.gz, Morien 2018) from the SILVA database (Quast et al. 2013). Taxonomy assignments were completed using the R package myTAI (Drost et al. 2018), which retrieves missing rank assignments based on the genus assignment from the NCBI database.

### Statistical analysis

The R statistical environment (R version 4.0.2) was used for data analysis (Team 2020) with the package ggplot2 for plotting (Wickham 2016). We inspected all data to check whether they satisfied normality assumptions using residual diagnostic plots following Fahrmeir et al. (2013) and applied data transformation if necessary (qPCR data were log-transformed). Root length colonization (%) and ratio AMF/plant gene were analyzed using two-way analysis of variance with a model including P supply, *Petunia* line and interaction of P supply and *Petunia* line. Phyloseq was used to analyze the ASV table (McMurdie and Holmes 2013). Rarefaction curves were prepared with vegan::rarecurve (Oksanen et al. 2018). Phyloseq::psmelt was used to prepare data for the taxonomy barplot constructed with ggplot2 (Wickham 2016). All code is available under https://github.com/PMI-Basel/Bodenhausen_et_al_AMF_qPCR.

## Results

### In silico primer validation

**Fig. 1:**
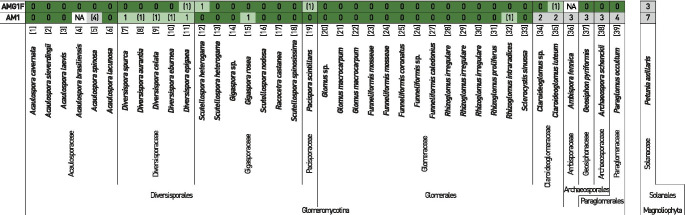
In silico analysis of PCR primer annealing sites across AMF species. The annealing sites of the PCR primers AMG1F and AM1 were inspected in the reference sequence set of AMF species (see “Material and methods”). The AMF species are sorted by consensus sequences ID ([1] to [39], indicated in square brackets above the species name) and grouped at order and family ranks. The detailed alignment of both PCR primers, of which the number of (partial) mismatches was derived, is shown in Fig. S1. As “partial mismatches” (number is indicated with brackets) we refer to primer bases that align to one of the nucleotide variants of wobble bases present at the annealing site. High sequence variability in consensus sequences [4] and [36] precluded alignments; indicated as “NA.” Annealing sites in *Petunia axillaris* were also analyzed as a plant out-group. Colors: dark green, perfect match; light green, 1 mismatch; grey, > 2 mismatches

First, we validated the “taxonomic breadth” of the AMF primers: do AMG1F and AM1 match the majority of species of the Glomeromycota? For this, we inspected the annealing sites of the qPCR primers in sequences of the phylogenetic reference set of AMF (Krüger et al. 2012). We also included the 18S rRNA gene sequence of *Petunia axillaris* as a plant out-group to confirm the specificity to AMF. The forward primer AMG1F perfectly matches to 34 of the 39 AMF species (Fig. S1), has one partial mismatch to 3 AMF species, and only one mismatch to *Scutellospora heterogama* (consensus sequence 12; Fig. 1). The 3 AMF species with partial mismatches have wobble bases at the primer annealing site but one of the nucleotide variants matches to the forward primer AMG1F (Fig. S1). *Ambispora fennica* (consensus sequence 36) and *Acaulospora brasilensis* (consensus sequence 4) could not be tested for annealing sites because of high sequence variability in the reference data.

In contrast to the forward PCR primer, the reverse PCR primer AM1 covers fewer AMF species with perfect sequence matches. AM1 perfectly matches 24 of the 39 AMF species, 4 AMF species have a partial mismatch (wobble nucleotide variants match to the reverse primer sequence), and 3 AMF species showed 1 mismatch (Figs. 1 and S1). Of note, all single mismatches to AM1 are in the middle of the primer annealing site and might still allow binding of the primer (Fig. S1). However, we found 7 of the 39 AMF species to have 2 or more mismatches at the annealing site, suggesting that these AMF species would not be amplified by AM1. We noticed an overall taxonomic signature in the distribution of mismatches which suggests AM1 to detect the *Acaulosporaceae, Gigasporaceae*, *Pacisporaceae*, and most *Glomeraceae*, probably also the *Diversisporaceae* but not the most ancestral AMF lineages of the *Claroideoglomeraceae*, *Archeosporales*, and *Paraglomerales* (Fig. 1). In these latter AMF species (consensus sequences 34 to 39), the non-matching nucleotides are located towards the 3′-end of the primer, which typically reduces the annealing efficiency (Dieffenbach et al. 1993). This suggests that a PCR product of the primers AMG1F and AM1 will not cover these ancestral AMF species. While this can be problematic for certain sample types and environments, analysis of plant roots should be less affected as sequences from these orders were rarely found in plant roots (Schlaeppi et al. 2016). Moreover, it is well known that these ancestral AMF species stain poorly under the microscope when using trypan blue or ink (Oehl et al. 2011) and as such microscopy also might not detect them.

The analysis of potential annealing sites in the 18S rRNA gene of *Petunia axillaris* revealed 3 and 7 mismatches for AMG1F and AM1, respectively (Figs. 1 and S1). This suggests an unlikely annealing of the PCR primers AMG1F and AM1 to DNA of *Petunia*. Altogether, this in silico analysis shows that the qPCR primers AMG1F and AM1 should cover most AMF species present in plant roots without amplifying host DNA.

### Experimental validation with microscopic analysis

After validating the primers in silico*,* we verified the relative qPCR method experimentally on DNA of plant root samples. We were interested to test whether the qPCR method can discriminate quantitative differences of AMF colonization and how the new method compares to traditional microscopy. We made use of a previously published experiment in which we measured a range from highly abundant to very low AMF root colonization using traditional microscopy (Bodenhausen et al. 2019). For biological validation of the qPCR method, we took advantage of the available DNA extracts and the previously determined levels of AMF colonization. In addition to Bodenhausen et al. (2019), we included new data from a second *Petunia* line (W115), which is a background for many mutants in *Petunia* research and was grown in the same pot experiment at the time but not included in the previous publication.

**Fig. 2:**
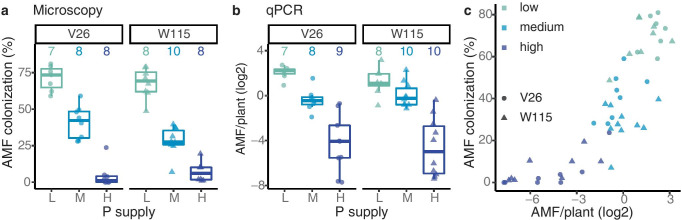
Microscopic and qPCR analyses of AMF root colonization. The two *Petunia* lines V26 and W115 were grown in pots supplied with three levels of phosphate fertilizer: low (L: 0.03 mM KH_2_PO_4_, medium (M: 1 mM KH_2_PO_4_), and high (H: 5 mM KH_2_PO_4_). **a** AMF root colonization was assessed by microscopy with the magnified intersection method on 100 intersections per sample. The data with *Petunia* line V26 were previously reported in (Bodenhausen et al. 2019). **b** DNA extracts from the same plants were used for qPCR analysis. The AMF signal, derived from qPCR primers AMG1F and AM1, was normalized relative to the plant signal of the glyceraldehyde-3-phosphate dehydrogenase (GAPDH). The number of plants for each treatment is shown at the top. **c** Relationship between AMF root colonization as assessed with traditional microscopy (ordinate, data of panel **a**) and relative qPCR (abscissa, data of panel **b**)

The quantification of AMF root colonization with traditional microscopy revealed that roots of both *Petunia* lines were abundantly colonized by AM fungi under low P conditions and that AMF root colonization decreased with increasing P supply (Fig. 2a). AMF root colonization as measured by microscopy was significantly affected by P supply (*p* < 0.001), with no evidence for an effect of *Petunia* line (*p* = 0.0737) or interaction of P supply and plant line (*p* = 0.0578).

We performed the relative qPCR with the available DNA extracts of the *Petunia* V26 and W115 root samples. For each extract we determined the level of AMF colonization using the qPCR primers AMG1F and AM1, and we normalized the AMF signal with the signal measured for the plant glyceraldehyde-3-phosphate dehydrogenase (GAPDH). The plant primers designed in this study amplified the GAPDH gene in both *Petunia* lines with the same efficiency (V26: 1.97 ± 0.007, *n* = 24, and W115: 1.97 ± 0.010, *n* = 28, mean efficiency ± s.e.m.; *T* test, *p* = 0.973). The quantification of AMF root colonization with the relative qPCR method confirmed that *Petunia* roots were abundantly colonized by AMF under low P and that the level of root colonization decreased with increasing P supply (Fig. 2b). AMF root colonization when measured by qPCR was significantly affected by P supply (*p* < 0.001) but did not differ by *Petunia* lines (*p* = 0.682) nor the interaction of the two (*p* = 0.596).

For each sample, we paired the data and examined the relationship between microscopy (Fig. 2a) and qPCR (Fig. 2b) using correlation analysis. The two methods show a strong positive correlation (Spearman correlation = 0.875, *p* < 0.001; Fig. 2c) revealing that AMF abundances agree sample-to-sample whether measured by microscopy or by qPCR. We noticed, however, that the relationship is not linear: at low levels of AMF colonization, the microscopy method is bounded by zero. On the other hand, the qPCR method can detect small differences in low levels of AMF colonization. The scatterplot suggests that qPCR offers improved resolution at low levels of AMF colonization compared with microscopy.

### Amplicon validation by sequencing

Next, we validated whether the qPCR primers AMG1F and AM1 indeed amplified sequences of AMF species and whether they avoided amplifying plant DNA. We used Illumina’s MiSeq instrument to sequence amplicons of these qPCR primers. For this analysis, we barcoded qPCR amplicons of the *Petunia* line V26 (4 biological replicates for low P supply and 4 replicates for high P supply), and as a negative control for primer specificity (*Arabidopsis* does not form symbiosis with AMF), we included root samples from *Arabidopsis* plants from the same pot experiment (Bodenhausen et al. 2019). Of note, the primers AMG1F and AM1 do produce an amplicon from *Arabidopsis* root DNA, which occurs at similar “late” PCR cycles (reflecting low levels of template DNA) as the *Petunia* plants that were very little colonized by AMF (high P supply; Fig. S2).

**Fig. 3:**
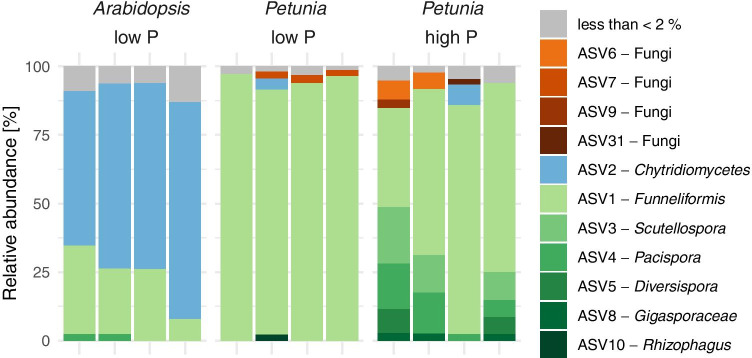
Taxonomic composition of PCR products amplified with AMG1F and AM1 revealed by MiSeq sequencing. The DNA of root samples from a previously published pot experiment (Bodenhausen et al. 2019) were amplified for qPCR and afterwards barcoded for MiSeq sequencing. Bars show relative abundance (in %) of sequence groups (amplicon sequence variants, ASVs) in each replicate sample. ASVs with more than 2% relative abundance in a sample are colored as follows: ASVs with best taxonomic assignment “kingdom = Fungi” are colored in shades of brown, an ASV belonging to class of Chytridiomycetes is blue and all ASVs belonging to the phylum Glomeromycota are colored in shades of green. ASVs with less than 2% relative abundance are in grey

We obtained a total of 167,145 high-quality sequences ranging from 7497 to 20,924 sequences per sample. Sequences were clustered into ASVs (amplicon sequence variants), and singletons were removed. Altogether, we obtained 349 ASVs with a range from 6 to 76 ASVs per sample. Rarefaction analysis reveals that the population of amplicon molecules has been sufficiently sampled at the obtained sequencing depth (Fig. S3). *Arabidopsis* samples have the highest numbers of ASVs followed by *Petunia* samples grown under high P and finally low P supplementation. The main rational for this sequencing analysis was to inspect the taxonomic profile of the population of molecules in the qPCR amplicons. As expected for plants grown in a pot experiment with field soil, and as suggested by the in silico primer analysis (Figs. 1 and S1), we found the large majority of sequences in the PCR amplicons of *Petunia* plants to be derived from Glomeromycota (Fig. 3). We identified the AM fungus genus *Funneliformis* to dominate in roots of *Petunia* plants grown under low P supplementation, whereas additional AMF fungi including those in the genera *Scutellospora*, *Pacispora*, and *Diversispora* were found in *Petunia* roots from high P supplementation. The low resolution of the taxonomic assignment of most ASVs is explained by the short length of the qPCR fragment being sequenced. Interestingly, in non-mycorrhizal *Arabidopsis* root samples, AMG1F and AM1 mainly detected *Chytridiomycetes* fungi and *Funneliformis* at a low level, consistent with previous reports of AMF growth along the roots of *Arabidopsis* plants (Veiga et al. 2013; Cosme et al. 2018).

Detection of the *Chytridiomycetes* in *Arabidopsis* raised the question whether other fungi might be amplified by this primer pair. We queried the SILVA database with our primer pair and found that of the 41,729 species in the database, only Glomeromycota fully match the tested primers (Fig. S4). Three or more mismatches will result in the amplification of fungi from other phyla like Ascomycota or Basidiomycota. In conclusion, AMG1F and AM1 primers are specific to Glomeromycota members.

## Discussion

Our motivation for this study was to develop a qPCR method to quantify AMF root colonization in a more high-throughput and less operator-dependent manner than classical microscopy. Our starting point was the qPCR primers developed by Hewins et al. (2015). First, we confirmed in silico their specificity to most AMF species (Fig. 1). We then validated their suitability to discriminate quantitative differences of AMF root colonization in a real experiment with *Petunia* plants. We demonstrated that AMF quantification with these qPCR primers is consistent with traditional microscopic analysis (Fig. 2). Of note, we normalized the quantification of the AMF qPCR signal relative to a plant qPCR signal, revealing that relative qPCR quantification works for quantification of root colonization. Finally, we validated using MiSeq sequencing that the qPCR primers produce AMF amplicons for mycorrhizal *Petunia* plants (Fig. 3). Our approach of relative qPCR with the primer pair AMG1F and AM1 presents an alternative molecular method to quantify AMF root colonization compared with traditional microscopy.

### Specificity

The advantage of the primer pair AMG1F and AM1 is that they amplify a wide range of species of the Glomeromycota. Our in silico analysis suggests this primer pair covers the *Acaulosporaceae*, *Gigasporaceae*, *Pacisporaceae*, most *Glomeraceae*, and probably also the *Diversisporaceae* but not the ancestral AMF lineages of the *Claroideoglomeraceae*, *Archeosporales*, and *Paraglomerales* (Fig. 1). Note that these ancestral AMF lineages are poorly visible under the microscope and as such also cannot be quantified using microscopy (Oehl et al. 2011). Broad taxonomic coverage of a wide range of AMF is a prerequisite to quantify the whole mycorrhizal community in different root samples, for example, from plants growing under different treatments or environments. These primers are different from qPCR primer pairs that were designed to specifically amplify a single AMF species (Alkan et al. 2004, 2006; Isayenkov et al. 2004; Jansa et al. 2008; Gamper et al. 2008; Thonar et al. 2012; Knegt et al. 2016), which are useful for research questions that require the quantification of colonization after inoculation with that single species.

In addition to covering a broad range of AMF species of the Glomeromycota, the primer pair AMG1F and AM1 is expected to avoid amplification of DNA from *Petunia*, as suggested by our in silico analysis (Fig. 1). We confirmed this prediction by sequencing qPCR products (Fig. 3). Our work with *Petunia* is consistent with an earlier validation by Hewins et al. (2015) that used Sanger sequencing to sequence qPCR amplicons from colonized wild leek. They sequenced 47 clones and found that all of them matched AMF, while none of them matched the plant host. The inclusion of non-mycorrhizal *Arabidopsis* as a negative control in our primer tests revealed that the PCR amplicon is formed in “late” PCR cycles (Fig. S2) and that it comprises mainly non-Glomeromycota fungi (Fig. 3). The PCR product of Petunia plants that were little colonized by AMF (high P supply) also forms at similarly “late” PCR cycles, but in that case this primer pair amplified mostly Glomeromycota sequences. Together this suggests for mycorrhizal plant species that AMG1F and AM1 primers specifically amplify Glomeromycota members even at low levels of AMF colonization. This observation is supported by the in silico analysis with the SILVA database which reveals that specific amplification is expected up to two mismatches to these primers (Fig. S4). Therefore, we think that the high level of detected Chytridiomycetes in the non-mycorrhizal *Arabidopsis* samples is likely because of abundant *Olpidium brassicae* (classified as *Chytridium* depending on the taxonomy and database, Lay et al. 2018) as previously reported in these roots (Bodenhausen et al. 2019).

In summary, the primer pair AMG1F and AM1 satisfies the requirement of covering the majority of AMF without amplifying host DNA which is necessary for a molecular method that quantifies total AMF root colonization.

### Independent method validation

A further requirement of a molecular method that quantifies AMF root colonization is that it should recapitulate the observations and findings from classical microscopy. In this study, we compared the relative qPCR approach to the traditional microscopy method, and we discussed the strengths and weaknesses of both methods (Table 1). We found good agreement in sample-to-sample comparisons of the microscopy quantifications with the results from relative qPCR (Fig. 2). The major difference was that qPCR showed less agreement at low root colonization levels than at high root colonization. This could be due to stochastic amplification during PCR at low DNA concentration. On the other hand, it could be due to enhanced resolution of qPCR at low colonization. For this reason, qPCR potentially can differentiate better between different intensities of colonization (as quantified by Trouvelot et al. 1986) compared with the classical microscopy method (as quantified by McGonigle et al. 1990). Independent method validations were successful for qPCR approaches that target single AMF species (Alkan et al. 2004; Isayenkov et al. 2004; Gamper et al. 2008; Werner and Kiers 2015), and here we show this is also true for the primer pair AMG1F and AM1, which depicts almost the entire AMF community.

**Table 1:** Advantages and disadvantages of qPCR and microscopy methods

	Microscopy	qPCR
Advantages	• Visualization of fungal structures under “real world” conditions • Widely used and applicable to a wide range of plant species • Quantification of different structures (hyphae, vesicles, arbuscules) • Provides an impression of AMF biology inside plant roots (morphology, life history) • Cost-effective regarding consumables and little infrastructure needs	• Scalable • Independent of the researcher • Comparable across different laboratories • Probably also applicable to old roots or roots that are difficult to stain (e.g., very young roots or thick roots) • High resolution at low levels of AMF colonization
Disadvantages	• Time-consuming • Observer-dependent • Difficult for thick or old plant roots • A new protocol (staining time etc.) must be developed for each plant species	• Different structures might contain different amount of DNA (e.g., AMF species that produce intraradical spores) • Relies on costly consumables and infrastructure (qPCR machine) • Tested so far for wild leek (*Allium tricoccum*) and *Petunia*. Applicability to other plant species needs validation

The main advantage of the qPCR method compared with the traditional microscopy method is that results are not biased by observers. Quantitative PCR permits determination of the overall level of AMF root colonization, but it does not permit quantification of different fungal structures (e.g., arbuscules, vesicles, or hyphae), which give insights into fungal physiology, as can be done with microscopy, nor does it permit distinction between frequency and intensity of colonization (Trouvelot et al. 1986). On the other hand, beginners or experienced researchers may differ in their recognition of different fungal structures. Even in the original article describing the magnified intersection method (McGonigle et al. 1990), the authors advised caution by stating “Most observers either overestimated or underestimated both proportions. By being consistent in this way an observer will correctly detect relative levels of colonization across experimental treatments, but these data will be observer-dependent and so should not be directly compared across experiments conducted by different researchers”. Because no individual enumeration of fungal structures is performed, qPCR results can be compared among different experiments even if different people processed the samples.

We see the scalability of the qPCR approach as a further advantage because many samples can be analyzed in relatively little time. We illustrate the time needs of both methods with the example of the pot experiment we analyzed (Table S2): For microscopy, samples need to be stained (ca. 3 h for 20 samples), then tiny root fragments need to be carefully mounted on a microscope slide (ca. 10 min per sample), finally at least 100 intersections need to be counted for each sample (ca. 15 min per sample). For the qPCR method, manual DNA extraction for 20 samples takes maximally 2 h, and 15 samples can be analyzed in one qPCR run, using a 96-well plate system and two primer pairs in triplicates, leading to ca. 3 h/run for preparation and analysis. Based on these rough estimates, the qPCR method takes about 2 times less time than the traditional microscopy method (Table S2).

In conclusion, a qPCR method that quantifies AMF root colonization is attractive compared with classical microscopy because the results are not observer dependent and because it saves time.

### Advantages of relative normalization

The qPCR primer pair AMG1F and AM1 was previously utilized for absolute quantification based on a standard curve (Hewins et al. 2015). In this study, we show that these primers also function to quantify AMF in *Petunia* roots with relative data normalization. We do not aim to directly compare absolute vs. relative data normalizations because both approaches have advantages and disadvantages depending on the research question and needs, so the choice of method depends on pragmatic reasons. For example, absolute quantification is appropriate for single AMF species analyses or when comparing colonization across different plant species. However, for multi-species quantification and comparative research questions—e.g., comparisons between treatments or plant genotypes—relative quantification has advantages related to the normalization of starting material and the nature of the DNA standard which are described in the following paragraphs.

The first advantage of the relative qPCR method is that there is no need of normalization with the starting material. Absolute quantification relies on highly accurate sample normalization, for example, of the amount of starting root material. Moreover, calculations can be affected by down-stream variation as, for instance, introduced by variable DNA extraction efficiencies of different sample types. A solution to this problem is to spike the sample with foreign DNA before DNA extraction and to perform a separate qPCR with a primer pair targeting this foreign DNA in order to quantity the DNA recovery factor (Thonar et al. 2012). Therefore, an advantage of relative quantification is that there is no need for normalization of the starting material or performing corrections for extraction recovery.

The second advantage of the relative qPCR method is that no DNA standard is required. A representative standard DNA is virtually impossible when quantifying mixtures of unknown microbes in environmental samples (Brankatschk et al. 2012). Standard curves are typically derived from a single cloned AMF sequence, and this permits the accurate quantification of the single AMF species from which the sequence clone was derived. However, the qPCR signal of such a standard curve might differ greatly from the signal of AMF mixtures present in environmental samples. On the other hand, relative quantification requires twice as many qPCR reactions, which will become costly with many samples. Each DNA extract needs to be assessed for both the AMF and the plant targets so that half a qPCR run will be filled with the AMF and the other half with the plant gene reactions. Moreover, if quantifying AMF root colonization across several plant genotypes, one first needs to establish that the primers for the plant gene perfectly match the genomic sequences of the locus in all compared genotypes so that they will amplify all genotypes with the same efficiency. In our case, a first version of the designed primer of the pair targeting the plant had a mismatch to the target sequence of one of the *Petunia* genotypes, so we had to design a second version to amplify the GAPDH gene in both *Petunia* lines with the same efficiency. Similarly, if the method were to be used for other plant species, we recommend validating the “non-specificity” of the PCR primers AMG1F and AM1 to the rRNA gene sequences of the plant species of interest by sequencing PCR products.

## Conclusion

In conclusion, qPCR with the primer pair AMG1F and AM1, which cover the majority of AMF species without amplifying *Petunia* host DNA, presents a useful alternative method to quantify AMF root colonization compared with traditional microscopy. Relative qPCR versus a plant gene reliably quantifies AMF root colonization in a less operator-dependent manner and at the same time offers scalability to more high-throughput analyses than microscopy. This comes, however, at the expense of insights into fungal physiology by not scoring fungal structures and nor having visual proof that roots are really colonized. We invite researchers to test this qPCR method with other plant species and in other environments than we have done.

## Electronic supplementary material

Below is the link to the electronic supplementary material.

Supplementary file1 (PDF 299 KB)

## Data Availability

The raw sequencing data is available from the European Nucleotide Archive (http://www.ebi.ac.uk/ena) with the sample ID SAMEA103939171 under the study accession PRJEB20127.

## Code availability

https://github.com/PMI-Basel/Bodenhausen_et_al_AMF_qPCR
