# Supplementary material for: Relative qPCR to quantify colonization of plant roots by arbuscular mycorrhizal fungi
Source: Mycorrhiza. 2021 Jan 21;31(2):137–48. doi: 10.1007/s00572-020-01014-1 (PMC7910240; doi:10.1007/s00572-020-01014-1)
Supplement: Supplementary file 1 — Supplementary file1 (PDF 299 KB) [file 572_2020_1014_MOESM1_ESM.pdf]

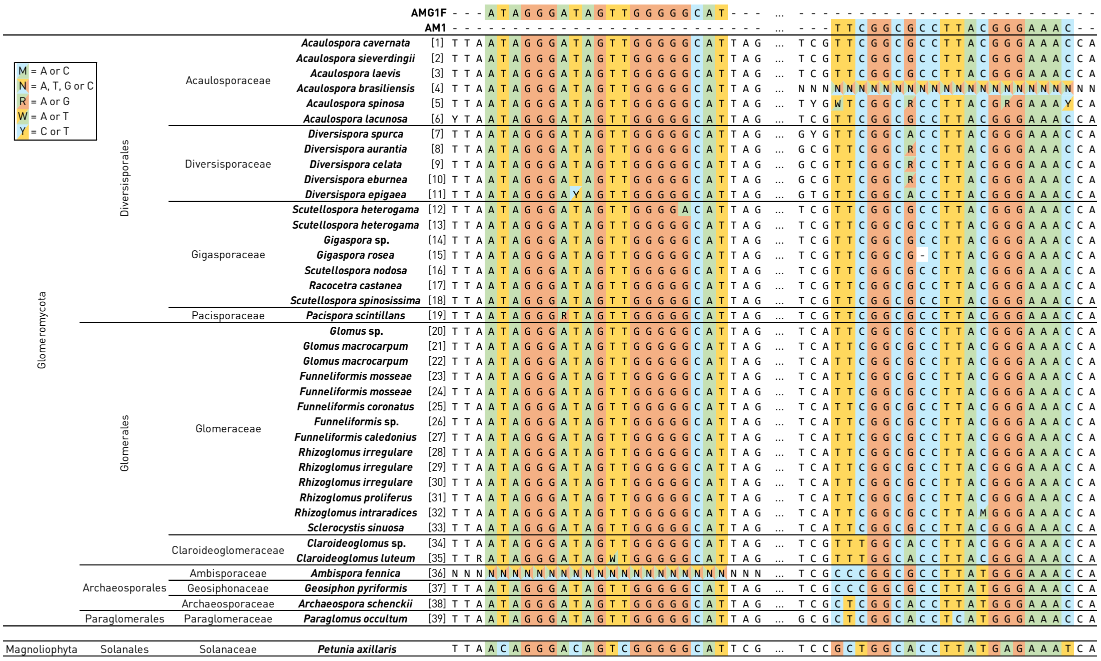

Supplementary Fig. S1 | Local alignment of AMG1F and AM1 to PCR primer annealing sites across AMF species

The annealing sites of the PCR primers AMG1F and AM1 were inspected in the reference sequence set of AMF species (see methods). The AMF species are sorted by consensus sequences ID ([1] to [39], indicated in square brackets following the species name) and grouped at order and family ranks. We display the detailed alignment of both PCR primers to the annealing sites in the consensus sequences, of which the number of (partial) mismatches is reported in **Fig. 1**. We defined 'partial mismatches' when a primer base still aligned to one of the nucleotide variants in wobble bases (see IUPAC code and colors in inset) present at the annealing site. High sequence variability in consensus sequences 4 and 36 precluded alignments. Annealing sites in *Petunia axillaris* also were analyzed as a plant out-group. Nucleotides are colored in green (A, adenine), yellow (T, thymine), red (G, guanine) and blue (C, cytosine).

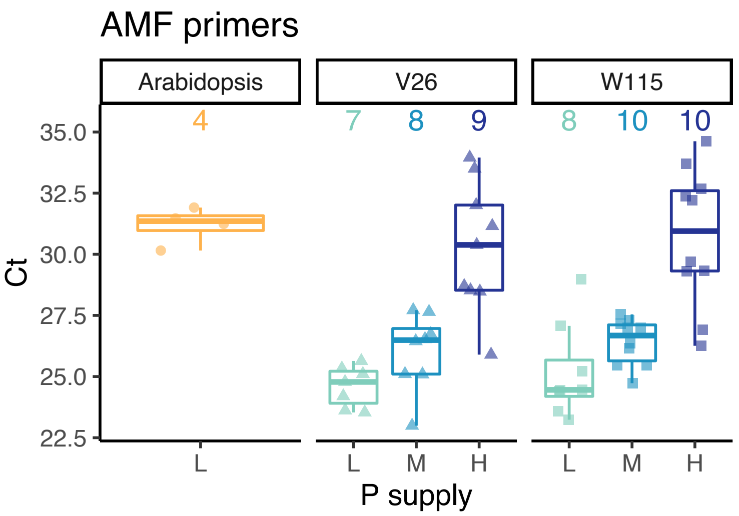

Supplementary Fig. S2 | Comparison of cycles to threshold values for amplicons generated with the primer pair AMG1F and AM1

The cycle threshold (Ct) values of the data presented in Fig. 2b (Petunia lines with low, medium and high P supply) is reported alongside the four Arabidopsis samples which were utilized as negative controls for the qPCR amplicon sequencing test. Ct values, which were set by default with linregPCR (Ruijter et al. 2009), are represented for the primer pair AMG1F and AM1.

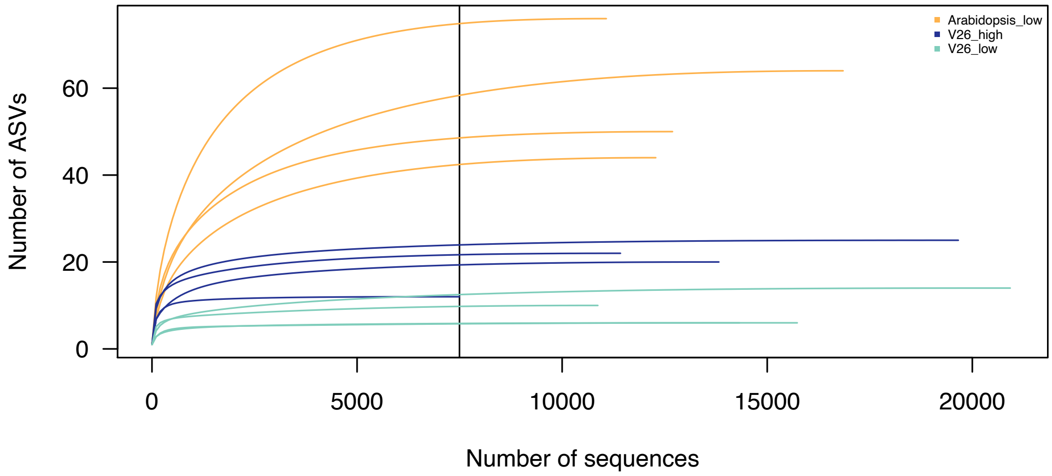

Supplementary Fig. S3 | Rarefaction analysis

This sampling intensity plot depicts the relationship of the number of detected ASVs depending on the sequencing depth. The curve reaches a plateau at the sequencing depth at which the sample was sufficiently deeply sequenced, i.e., all molecules in the population were covered. The vertical line marks the sample with the smallest number of sequences across all samples.

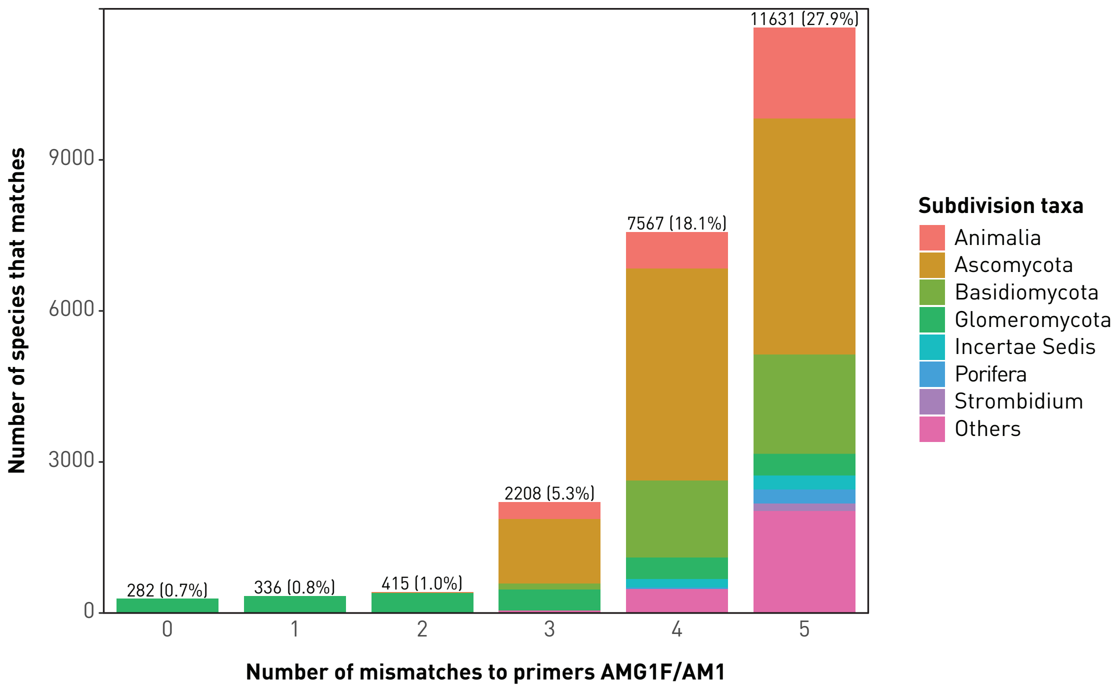

Supplementary Fig. S4 | Mismatch analysis of AMG1F and AM1 primers.
A global mismatch analysis was performed using https://www.arb-silva.de/search/testprime/. The number of species and their proportion (in parentheses), compared to the total number of species, are indicated on top of each bar.

Supplementary Table S1. Barcode to sample assignments

plant	treatment	replicate	forward_barcode_sequence	reverse_barcode_sequence
Arabidopsis	low	replicate2	TGGCTACA	GGCGAATA
Arabidopsis	low	replicate3	TCTAGGAG	GGCGAATA
Arabidopsis	low	replicate4	TAGTGCCA	GGCGAATA
Arabidopsis	low	replicate5	GTCTGAGT	GGCGAATA
V26	low	replicate7	AGATACGG	CTGCCATA
V26	low	replicate5	TGGCTACA	CTGCCATA
V26	low	replicate4	ATCCACGA	GATCAAGG
V26	low	replicate8	ATCCACGA	CTGCCATA
V26	high	replicate5	TCTAGGAG	GATCAAGG
V26	high	replicate7	GTCTGAGT	GATCAAGG
V26	high	replicate6	AGATACGG	GATCAAGG
V26	high	replicate1	TGGCTACA	GATCAAGG

Supplementary Table 2. Comparison of estimated time required to process samples by microscopy or qPCR

**MICROSCOPY**	*(49 samples)*	**qPCR**	*(49 samples)*
Staining (3 h/20 samples)*	7 hours	DNA (2 h/20 samples)	5 hours
Mounting (10 min/sample)	8 hours	qPCR (3 h/15 samples)	10 hours
Microscopy (15 min/sample)**	12 hours		
Total microscopy	27 hours	Total qPCR	15 hours

*The time required per sample depends on the host plant, age of the roots and whether plant roots are collected from a greenhouse experiment in sterilized soil (with reduced fungal diversity) or from field soil with many organisms colonizing the roots.

**Time needed for an experienced researcher is about 15 minutes per sample. For beginners, more time is required and a proper introduction by an experienced researcher helps to identify the appropriate fungal structures.
